# Governing global capital: How state capacity and climate policy credibility attract sustainable investment

**DOI:** 10.1093/pnasnexus/pgag315

**Published:** 2026-10-09

**Authors:** Huanhuan Zheng

**Affiliations:** Department of Public and International Affairs, City University of Hong Kong, 83 Tat Chee Ave, Kowloon Tong, Kowloon, Hong Kong SAR

**Keywords:** sustainable growth, ESG, climate policy, state capacity, regulatory state

## Abstract

Can governments steer global capital toward public goals in an era of market-driven finance? This paper examines how state capacity and institutional credibility enable governments to attract sustainable investment through climate policy. Using the adoption of climate policies across 94 economies, we apply a triple difference-in-differences design to distinguish the effects of policy announcements, implementation, and demonstrated performance. We find that announcements increase sustainable investment flows by 24 basis points per week even before policies take effect, while the effect rises to 45 basis points following implementation. Demonstrated policy performance further amplifies these flows: above-median improvements in emissions, renewable-energy generation, and green innovation attract an additional 13–16 basis points per week. In contrast, improvements in financial returns and risk do not explain these flows. The study bridges the literature on state capacity and sustainable finance, showing that credible, high-capacity governance is foundational for mobilizing private capital toward climate goals—a critical insight for designing effective climate governance.

Significance Statement Financing the transition to a net-zero economy requires trillions of dollars in private investment, which is far beyond what governments can fund alone. This study shows that credible climate policy can help attract that capital. Climate-policy announcements increase sustainable investment even before policies take effect, and the response nearly doubles after implementation, from 24 to 45 basis points per week. Governments that subsequently deliver stronger emissions reductions, renewable-energy expansion, and green innovation attract still more sustainable capital. These effects are not explained by higher financial returns or lower risk. Investors therefore reward not only policy commitments but also governments' capacity to implement them and deliver results. Effective climate governance thus serves simultaneously as environmental policy and strategy for mobilizing private capital.

## Introduction

Governments worldwide face a monumental governance challenge: how to finance the transition to a net-zero economy. With public budgets constrained, relying on state resources alone is insufficient to close the multi-trillion-dollar investment gap needed for green infrastructure and technology. This raises a central question in contemporary climate governance: can the state steer global capital toward public goals in an era of financial globalization? Building on the concept of the regulatory state (1), this paper examines how governments use climate policy not only as an environmental tool but also as a governance mechanism to mobilize international sustainable investment toward climate goals through the construction of institutional credibility.

While previous research has extensively documented the social and environmental benefits of climate policy—such as reductions in carbon emissions (2), increased renewable-energy adoption (3), and green innovation (4)—relatively little is known about how and why these policies attract private investment. Existing studies in international political economy and environmental finance emphasize policy adoption and design (5–7), yet they tend to treat policy credibility as static, rather than as a dynamic governance process that evolves through signaling, implementation, and demonstrated performance. Consequently, we lack systematic understanding of how governments build, institutionalize, and sustain credibility, and how these processes shape investor expectations and the allocation of global capital.

This paper addresses that gap by developing and testing a multi-stage theory of policy credibility. We conceptualize credibility as an evolving process: first signaled through policy announcements that communicate government intent, then reinforced through implementation that institutionalizes enforcement, and finally consolidated through demonstrated state capacity that delivers measurable climate outcomes. This dynamic view bridges governance and finance, highlighting how credible policy performance can transform domestic regulation into a magnet of international capital.

The rise of sustainable investing creates a pivotal opportunity for governments to leverage private finance for climate goals. Unlike traditional capital, sustainable investors explicitly seek environmental and social returns alongside financial performance. If policymakers understand what drives these flows, they can design more effective strategies to attract private investment into climate-aligned activities. We therefore ask: Do climate policies, such as carbon taxes and emissions trading systems (ETS), attract international sustainable investment? And more importantly for policy design, why do they succeed—is it by delivering tangible social and environmental outcomes or by improving financial incentives?

To answer these questions, we exploit the worldwide adoption of climate policies to evaluate their effects on international investment. If climate policy effectively advances mitigation goals, sustainable investors—who value social impact and policy credibility—should respond more strongly than traditional investors. Conversely, if climate policy merely improves expected returns or reduces market risk, both investment types should react similarly. By comparing sustainable and traditional investments within the same country and over time, our triple differences-in-differences (DiD) design differences out all economy-wide shocks—such as monetary policy or market-wide movements—that affect both types of investments alike, thereby identifying the unique effect of climate policy on sustainable investment. Drawing on weekly investment flows over two decades, this study provides international evidence that credible and effective climate governance can systematically attract the international private capital crucial for financing the green transition.

Our findings offer three key insights. First, climate policy is a powerful tool for attracting sustainable investment. The announcement of a new climate policy increases sustainable investment flows by about 23 percentage points per year, suggesting that clear government commitment can influence global capital allocation even before policies take effect. Second, the credibility of that commitment strengthens with implementation. Once climate policies are formally enforced, sustainable investment rises further, showing that investors respond not only to political intent but also to evidence that governments can follow through. Third, the magnitude of investment response depends on policy effectiveness. Sustainable investors channel more capital to countries where climate policies deliver tangible results—such as larger emission reductions, greater renewable-energy expansion, and stronger green innovation. These patterns indicate that investors reward credible and capable climate governance.

## Theory and hypothesis

The expanding literature on climate governance and sustainable finance has underscored the importance of credible policy commitments for mobilizing private capital, yet it remains unclear how policy credibility is constructed and transmitted to investors. Existing research focuses on policy adoption or design (2, 5–7), implicitly treating credibility as an inherent institutional feature. We instead conceptualize credibility as a dynamic process of institutional development, consistent with the shift from the interventionist to the regulatory state (1). In this mode, the state “steers” through rules, incentives, and information rather than “rows” through direct provision, but such steering works only when markets perceive government commitments as credible.

This view builds on foundational theories of policy credibility. Kydland and Prescott (8) and Barro and Gordon (9) show that credible policy requires governments to overcome time-inconsistency problems and convince markets that commitments will endure. Levy and Spiller (10) similarly argue that regulatory credibility depends on institutional constraints that limit opportunistic reversal. We extend these insights to climate governance, where credibility is especially difficult because policy commitments are long-term, politically contested, and dependent on state capacity.

We theorize policy credibility as a three-stage process of institutionalization: announcement, implementation, and demonstrated performance. Figure S1 illustrates this framework. The announcements provide the initial signal of government intent. Drawing on signaling theory (11, 12), we argue that climate-policy announcements operate as costly institutional commitments that reduce uncertainty and coordinate forward-looking sustainable investors. Implementation then converts expectations into enforceable constraints, embedding policy in bureaucratic routines and vested interests that make reversal politically and economically costly (13, 14). Finally, demonstrated performance validates state capacity and further strengthens credibility. Building on state capacity theory (15–17), we argue that observable outcomes—such as emission reductions, renewable-energy expansion, and green innovation—provide the strongest credibility signal by converting institutional intent into observable reliability. Investors, especially those with long horizons and climate mandates, interpret such performance as evidence of effective governance, lowering perceived policy and execution risk.

### Policy announcements as credible institutional signals

Governments have shifted from an interventionist state that “rows” through direct provision, ownership, and spending to a regulatory state that “steers” by setting rules, prices, and disclosure requirements to guide market actors (1). Climate policies such as ETS and carbon taxes exemplify this logic: governments make emissions costly and clean investment more attractive. Announcing such policies signals commitment to climate objectives and reduces uncertainty about future regulation. This signal mobilizes private capital only when investors view it as credible—that is, when they expect the policy path to be delivered and sustained. Although Majone's framework was developed mainly in advanced democracies, this credibility mechanism should extend across regime types because investors evaluate observable commitments, implementation capacity, and policy performance rather than democratic institutions alone. Democracies may build credibility through accountability and legal constraints, while hybrid and authoritarian regimes may do so through administrative control, coordination, reputation, and enforcement capacity. Thus, regime type may condition the strength of the effect, but it should not negate the mechanism.

Climate-policy announcements are credible institutional signals because they are costly and reputation-binding, not merely symbolic. Before confirming an ETS or carbon tax, governments typically incur political and administrative costs, including legislative preparation, coalition building, and bureaucratic planning. Announcement raises the cost of reversal by inviting scrutiny from media, opposition parties, civil society, and market actors, creating reputational liabilities if governments fail to follow through (1). Because credibility increases with the cost of reneging, announcements reveal governmental intent (12) and establish expectations under institutional constraints (11). They convert uncertainty about future regulation into public information that investors can act on before implementation.

Credible climate-policy announcements attract sustainable investors through three governance functions. First, they open a policy window by committing political attention and administrative resources to climate objectives (18). These high-salience, low-search-cost cues align with sustainable investors' mandates, reduce climate-policy uncertainty, and signal support for low-carbon assets. Second, announcements coordinate expectations by creating common knowledge about future policy direction, lowering coordination costs and allowing forward-looking investors to adjust strategies before regulatory change (19, 20). Third, they build “green credibility” in transnational arenas by signaling alignment with global climate norms, enabling policy learning, and strengthening reputations among international audiences (13, 21). This parallels other reputational mechanisms: fiscal reforms can bolster credibility within monetary unions (22), credit ratings shape states' capital-market access (23), and credible climate commitments communicate reliability to markets and peers. As states compete for capital from powerful international constituencies (24), such signals should be especially salient to sustainable investors.

Governance theory explains why announcements may matter through credible signaling, agenda-setting, and discursive steering, but it remains unclear whether preimplementation domestic announcements systematically reallocate international private capital. We address this gap by using high-frequency sustainable fund flows as a revealed-preference measure of perceived credibility. If climate-policy announcements are costly, reputation-binding signals that create common knowledge and reduce policy uncertainty, they should attract climate-mandated sustainable investment even before enforcement.

H1 (Institutional signaling): The announcement of a climate policy, as an institutional signal of intent, will increase sustainable investment flows.

### Implementation as the institutional deepening of credibility

Climate-policy announcements initiate credibility by signaling commitment; implementation deepens it by converting expectations into enforceable institutional constraints. This distinction parallels the difference between policy commitment and policy capacity: costly signals create initial credibility, but institutional execution consolidates it (7, 16).

Implementation should further increase a jurisdiction's attractiveness to sustainable investors for three reasons. First, it turns political commitment into enforceable rules, reducing regulatory and policy uncertainty (11). Second, functioning policies strengthen reputations for reliability and institutional learning, lowering perceived governance risk (13). Third, an operational carbon market embeds climate policy in administrative routines and vested interests—regulated firms, financial intermediaries, and bureaucratic agencies—making reversal more politically and economically costly and reinforcing expectations of policy stability (14).

Thus, as climate policy moves from announcement to implementation, credibility becomes more institutionalized, amplifying its effect on sustainable investment.

H2 (Implementation amplification effect): The positive effect of climate policy on sustainable investment is stronger after implementation than after announcement.

### Demonstrated state capacity as a credibility mechanism

Beyond announcements and formal implementation, the credibility of climate policy ultimately depends on demonstrated performance. Investors look not only for promises or legal enactments but also for evidence that policies deliver results. Observable success—such as sustained reductions in carbon emissions, expansion of renewable energy, or growth in green innovation—signals high administrative, regulatory, and political capacity. These outcomes provide public, verifiable indicators of state effectiveness and generate credible signals of competent, rule-based governance (15–17).

Investors should reward demonstrated competence because it lowers perceived policy and execution risk and signals a more stable regulatory environment for long-term investment. This mechanism is especially salient for sustainable investors, whose mandates are tied directly to climate outcomes. When outcomes are credible and reputational cues are clear, audiences update beliefs more strongly (25, 26). Jurisdictions with more effective climate policies should therefore appear more capable and durable, attracting stronger and more sustained capital inflows. Over time, realized climate performance may also become a reputational asset in markets and transnational networks, expanding access to sustainable finance and reinforcing policy diffusion (7).

This constitutes the state capacity channel: demonstrated policy performance deepens institutional credibility, thereby magnifying the positive effect of climate policy on sustainable investment.

H3 (The state capacity channel): The positive effect of climate policy on sustainable investment will be stronger for policies that demonstrate higher state effectiveness.

### Institutional primacy over financial incentives

The preceding discussion emphasizes the state capacity channel, through which effective governance amplifies the impact of climate policy on sustainable investment. A competing explanation is the financial channel. From this view, climate policy attracts capital not by signaling credible governance, but by changing expected returns and risks. Carbon prices, clean-energy subsidies, and tax incentives may raise the profitability of low-carbon assets relative to carbon-intensive ones, improve risk-adjusted returns, and attract investors for purely financial reasons.

We argue that this financial channel is secondary to the credibility-based state capacity channel for two reasons. First, financial incentives are often temporary, market-sensitive, and insufficient to anchor long-term capital without credible institutions that can sustain policy commitments. If the state lacks capacity to enforce and maintain rules, investors will discount financial gains as short-lived or reversible (7). Second, sustainable investors differ from conventional investors: their mandates reflect a “logic of appropriateness” grounded in social and environmental objectives, not only a “logic of consequences” based on expected returns (27). They should therefore respond more strongly to policy effectiveness and institutional reliability than to marginal financial gains.

Thus, we expect the effect of climate policy on sustainable investment to be driven primarily by credible state capacity and demonstrated policy effectiveness, rather than by improved returns or lower risk alone.

H4 (Institutional vs. financial mechanisms): The effect of climate policy will be primarily driven by the state capacity channel, rather than by direct financial incentives.

## Results

### Summary statistics

Figure 1A shows that global sustainable investment rose from $11 million in 2001 to almost $2 trillion in 2021, an 83% compound annual growth rate—more than triple the growth of traditional investments (26%). Its share of mutual fund assets under management (AUM) increased from 5 to 8% over the same period. As Fig. 1B shows, this share is especially high in European countries with generally stricter carbon regulations, reaching 25% in Sweden, 20% in Spain, and 17% in both Austria and Switzerland in 2021.

**Figure 1:**
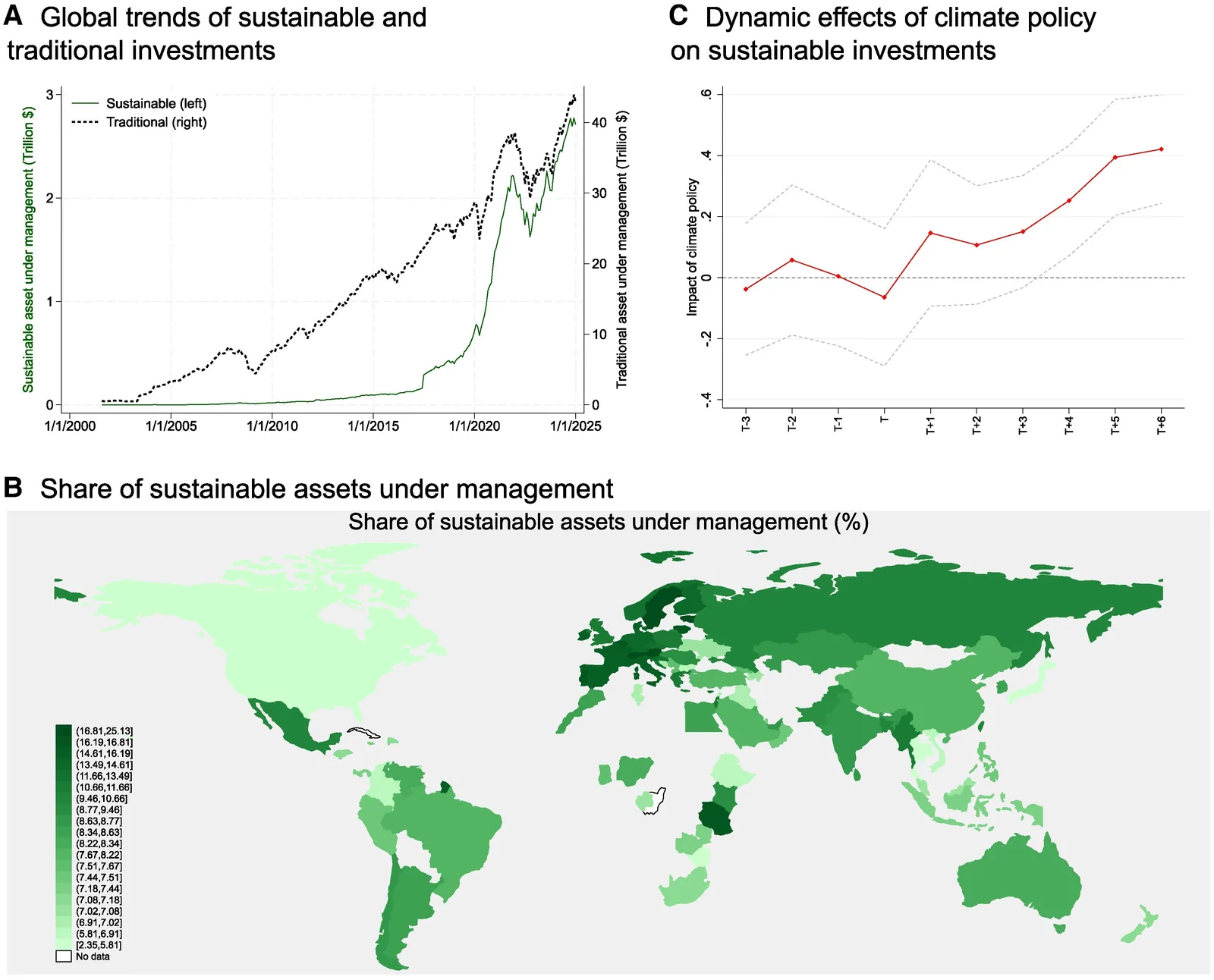
Trends and distribution of sustainable investments and their response to climate policy. The solid and dashed lines in (A) plot the stock of sustainable and traditional investments worldwide in trillion US$ from 2000 to 2021. (B) The share of the stock of sustainable investments to the sum of the stock of both sustainable and traditional investments in each economy as of 2021, with darker color representing greater share. (C) The difference between sustainable and traditional investments from 3 years before the announcement of the climate policy to 6 years after, in solid line. The dashed lines are the 95% CIs.

Table S1 compares weekly sustainable and traditional investment flows before and after climate-policy announcements. Sustainable investments already exceed traditional investments before announcement, and this gap widens by 3.8 basis points per week, or 2 percentage points annually, afterward. Although sustainable investments initially have lower returns and higher risk, both gaps narrow significantly postannouncement. Overall, the descriptive evidence suggests that climate policy promotes sustainable investment while improving its risk-return profile.

### Baseline result

To formally evaluate the impact of climate policy on sustainable investment, we employ a triple-DiD approach comparing sustainable and traditional investments within the same economy before and after climate-policy announcement (Eq. 1). Table 1, column 1 shows that the weekly gap between sustainable and traditional investment flows rises by 39 basis points after announcement. This implies a 23-percentage-point annual increase in sustainable investment, compounded over 52 weeks. Given an average global stock of $7.7 trillion in sustainable AUM in our sample, this effect corresponds to an additional US$1.8 trillion in annual private capital flows for climate mitigation and adaptation—∼45% of the IEA's estimated US$4 trillion annual clean-energy investment gap needed to achieve net-zero emissions by 2050.

**Table 1:** Impacts of climate policy on sustainable investments.

	(1)	(2)	(3)	(4)
Sample	Full	Sustainable only	Traditional only	Full
CP		0.047**	−0.028	
		(0.020)	(0.018)	
SRI × CP	0.388***			0.242***
	(0.042)			(0.091)
SRI × CP^Imp^				0.208**
				(0.091)
Constant	0.075***	0.239***	0.073***	0.066***
	(0.008)	(0.009)	(0.007)	(0.009)
Observations	276,961	129,749	156,799	276,961
*R* ^2^	0.412	0.474	0.232	0.413

The dependent variable is Investment, the weekly capital flows to either sustainable or traditional funds. SRI equals 1 for sustainable investments and 0 for traditional investments. CP equals 1 after the announcement of the climate policy, and 0 otherwise. The postimplementation dummy ${\mathrm{CP}}_{\;}^{\mathrm{Imp}}$ equals 1 after the official implementation of climate policy and 0 otherwise. Columns 1 and 4 are based on the full sample, while columns 2 and 3 utilize, respectively, the subsample of sustainable and traditional investments. All regressions control for sustainability-asset-economy, asset-time, and economy-time fixed effects. Heterogeneity robust SE clustered by economy is reported in the parenthesis. *** and ** denote the significance level at 1 and 5%, respectively.

We next examine whether this effect reflects growth in sustainable investment or a decline in traditional investment. In the sustainable-investment subsample, Table 1, column 2 shows a significant postannouncement increase relative to preannouncement levels. In contrast, Table 1, column 3 provides no statistical evidence that traditional investment changes significantly after announcement. Thus, the estimated effect is driven by growth in sustainable investment rather than a decline in traditional investment, supporting the difference-in-differences design.

The effect remains robust to alternative fixed effects, clustering and estimators (Table S2), separate analyses of equity and bond markets, and controls for additional variables (Table S3). We further show that the effect is stronger when carbon prices are higher and climate regulation covers a broader share of the economy, consistent with investors responding more to stringent and credible policy signals (Table S4).

### Announcement versus implementation effects

The baseline results capture the average postannouncement effect, but this may combine the signaling effect of announcement with the credibility gains from implementation. We further separate the postannouncement period into announcement and implementation phases. The announcement captures the initial credibility gained when governments publicly commit to future carbon regulation. Such costly, reputation-binding signals reduce uncertainty and prompt forward-looking investors to reallocate capital before enforcement, consistent with H1. Implementation then provides verifiable evidence of follow through, converting expectations into enforceable commitments and strengthening investor confidence, consistent with H2.

Estimating Eq. 2, Table 1, column 1 shows that climate-policy announcements increase sustainable investment even before implementation, supporting H1. After implementation, the effect rises by 45 basis points per week, 86% larger than the 24-basis-point announcement effect, consistent with H2. Investors therefore respond both to credible signaling at announcement and to institutional reinforcement at implementation. Distinguishing these stages is important, as ignoring announcement effects would underestimate the full impact of climate policy on sustainable investment.

### Dynamic effects

Figure 1C shows the dynamic effects of climate policy on sustainable investment from 3 years before to 6 years after announcement. Before the announcement, the gap between sustainable and traditional investments was economically small and statistically insignificant, supporting the parallel-trends assumption. After announcement, the gap increases persistently and eventually becomes statistically significant, with no reversal even 6 years later. This pattern reduces the likelihood that other factors drive the results and supports attributing the rise in sustainable investment to climate policy.

### Mechanism

Sustainable investors seek both environmental impact and financial returns. Climate policy may therefore attract sustainable capital through two channels. The state capacity channel predicts that measurable climate progress signals credible governance and durable policy commitment, attracting investors whose mandates value environmental outcomes. The financial channel predicts that climate policy attracts capital by improving the risk-return profile of green assets. We exploit cross-country variation in climate policy performance to test these mechanisms.

#### State capacity channel

We estimate Eq. 3 to test whether climate policies that deliver stronger mitigation outcomes attract more sustainable investment. Prior research shows that climate policy can reduce carbon emissions (2), promote renewable-energy generation when supported by public subsidy (3), and stimulate green innovation (4), although effectiveness varies across economies. Sustainable investors may not observe the institutional processes behind these outcomes, but they can infer state capacity from visible results: declining emissions, expanding renewable capacity, and green innovation. Because sustainable investors derive nonpecuniary value from environmental impact, they should respond more strongly when policies deliver tangible climate progress, as predicted by H3.

Table 2 supports this mechanism. Sustainable investment increases by 16 basis points per week (8 percentage points annually) when climate policies deliver above-median emissions reductions, by 13 basis points (7 percentage points annually) when renewable generation expands more rapidly, and by 16 basis points (9 percentage points annually) when green innovation grows more strongly. These results show that investors reward demonstrated performance, not merely policy intent or legal adoption. Emission cuts, renewable expansion, and green innovation serve as credible signals of state capacity, administrative competence, and policy durability, supporting H3.

**Table 2:** The state capacity channel

	(1)	(2)	(3)
SRI × CP	0.360***	0.370***	0.383***
	(0.051)	(0.053)	(0.048)
SRI × CP × D^Carbon^	0.105***		
	(0.039)		
SRI × CP × D^Renewable^		0.073**	
		(0.037)	
SRI × CP × D^Technology^			0.092**
			(0.038)
Observations	245,950	245,950	245,950
*R* ^2^	0.420	0.420	0.420

This table summarizes how sustainable investments respond to climate policies associated with different policy performance. The dependent variable is Investment, the weekly capital flows to either sustainable or traditional funds. The sustainable indicator SRI equals 1 for sustainable investments (treatment group), and 0 for traditional investments (control group). The posttreatment dummy CP equals 1 after the announcement of the climate policy, and 0 otherwise. The climate mitigation dummy variables $D_{\;}^{\mathrm{Carbon}}$, $D_{\;}^{\mathrm{Renewable}}$, and $D_{\;}^{\mathrm{Technology}}$ equal 1, respectively, when the reductions of carbon emissions, the improvements to renewable-energy generation and green technology innovations after the climate policy's introduction, relative to their prepolicy levels, are above the sample median, and 0 otherwise. All regressions control for sustainability-asset-economy, asset-time, and economy-time fixed effects. Heterogeneity robust SE clustered by economy is reported in the parenthesis. *** and ** denote the significance level at 1 and 5%, respectively.

Climate policy also continues to attract significant sustainable investment in economies with below-median effectiveness. This suggests that investors value the institutional commitment signaled by climate policy adoption itself, while stronger state performance substantially amplifies the effect.

#### Financial channel

To examine whether climate policy attracts sustainable investment through financial incentives, as opposed to credible governance performance, we first evaluate whether climate policy alters the risk-return profile of sustainable investments. Existing studies have shown that booming sustainable investments reduce green financing costs (28–30) and increase the risk premium required for climate risk exposure (31–34). Yet this literature focuses mainly on investor preferences, not on whether policy effectiveness itself improves sustainable asset performance. We extend it by testing how climate policy affects sustainable investment returns and risks.

Carbon pricing can raise the relative value of carbon-light firms by increasing costs for carbon-intensive firms (35–37). It may also create stranded-asset risks (38) and other hard-to-price uncertainties (39). Because investors price stranded-asset and regulatory risks (40), carbon-light firms face lower relative risk exposure (41). Climate policy may therefore raise returns and reduce risk for sustainable portfolios tilted toward carbon-light assets. Consistent with this possibility, Table 3 shows that sustainable investment returns increase by 12 basis points per week (7 percentage points annually, column 1), while return volatility falls by 26 basis points per week (15 percentage points annually, column 2).

**Table 3:** The financial incentive channel.

	(1)	(2)	(3)	(4)	(5)
	Return	Risk	Investment	Investment	Investment
SRI × CP	0.115***	−0.262***	0.373***	0.445***	0.431***
	(0.010)	(0.039)	(0.039)	(0.037)	(0.034)
SRI × CP × D^Return^			0.032*		0.026
			(0.016)		(0.016)
SRI × CP × D^Risk^				−0.094***	−0.092***
				(0.024)	(0.024)
Observations	276,961	276,625	276,961	276,961	276,961
*R* ^2^	0.912	0.929	0.412	0.413	0.413

This table summarizes how sustainable investments respond to climate policies associated with different financial outcomes. The dependent variables are Return, the weekly profit normalized by total asset under management of either sustainable or traditional funds in each asset class of each economy (column 1), Risk, the standard deviation of the investment returns in the past 52 weeks (column 2), and Investment, the weekly capital flows to either sustainable or traditional funds (columns 3–5). The sustainable indicator SRI equals 1 for sustainable investments (treatment group), and 0 for traditional investments (control group). The posttreatment dummy CP equals 1 after the announcement of the climate policy, and 0 otherwise. The financial dummy variables $D_{\;}^{\mathrm{Return}}$ and $D_{\;}^{\mathrm{Risk}}$ equal 1, respectively, when the return improvements and risk reductions associated with climate policies are above the sample median, and 0 otherwise. All regressions control for sustainability-asset-economy, asset-time, and economy-time fixed effects. Heterogeneity robust SE clustered by economy is reported in the parenthesis. *** and * denote the significance level at 1 and 10%, respectively.

If the financial channel drives sustainable investment, policies producing larger return gains or risk reductions should attract more capital. We therefore redefine the channel dummy using postpolicy return improvements and risk reductions, then re-estimate Eq. 3. Table 3, column 3 shows that above-median return improvements attract only 3 additional basis points per week, or 1.7 percentage points annually—economically small and statistically insignificant at the conventional 5% level. This weak relationship disappears after controlling for risk (column 5). Moreover, sustainable investment responds more strongly to policies with below-median risk reductions (column 4), contradicting the prediction that lower risk drives greater inflows. This suggests that sustainable investors respond more to credible climate commitments and demonstrated governance capacity, even where measured risk reductions are modest.

Thus, while climate policy improves sustainable investments' risk-return profile, these improvements do not explain the rise in sustainable capital. The evidence supports H4: sustainable investment is driven primarily by state capacity, while financial gains appear to be a consequence rather than the main mechanism.

## Discussion

This study shows that climate policy is not only an environmental instrument but also a governance mechanism for mobilizing private capital. Sustainable investment responds to a sequential process of credibility building: announcements signal commitment, implementation demonstrates follow through, and demonstrated performance—lower emissions, expanded renewable energy, and green innovation—validate state capacity. Investors distinguish not only between countries that adopt climate policies and those that do not, but also between governments that merely promise and those that deliver. Although climate policy also improves the risk-return profile of sustainable investment, these financial gains do not explain the observed capital response. What matters more is credible and demonstrated governance capacity.

This interpretation does not imply that climate policy originates independently of private interests. From a public-choice perspective, firms and investors may lobby for regulations that favor their assets, create barriers to entry, or raise competitors' costs; capital may therefore shape policy as well as respond to it. Our design does not identify the political origins of climate-policy adoption and cannot rule out such influence. It instead shows that, once policies are announced, investors respond increasingly as governments implement them and demonstrate effective performance. Public choice and state capacity are therefore complementary rather than competing perspectives: private interests may help shape which policies governments adopt, while state capacity and credibility help explain how capital responds as those policies are implemented and deliver results.

These findings carry three policy implications. First, governments should treat credibility as an asset: clear announcements, enforceable rules, and consistent implementation can reduce uncertainty and attract sustainable capital. Second, they should invest in administrative capacity, since investors reward governments that can translate commitments into measurable progress. Third, they should make climate performance transparent through credible reporting on emissions reductions, renewable-energy expansion, and green innovation. Visible performance turns policy success into a reputational advantage.

For fiscally constrained states, this offers a practical pathway to expand financing capacity without relying solely on public spending or subsidies. By leveraging institutional credibility, governments can align private finance with public climate goals. In this sense, effective climate governance creates both environmental and economic dividends: credibility and state capacity become forms of capital in financing decarbonization.

## Materials and methods

### Data

#### International investments

We use weekly international sustainable investment data from Emerging Portfolio Fund Research (EPFR), which tracks mutual funds integrating criteria of environmental, social, and governance (ESG) or socially responsible investing (SRI). Funds labeled as sustainable undergo ESG or SRI screening and emphasize social welfare alongside financial returns. EPFR records AUM, capital flows, and returns by economy, separately for equity and bond markets. Comparable data are available for traditional funds. To ensure cross-country and cross-asset comparability, flows are normalized by lagged AUM. Specifically, investment flows are measured as weekly capital flows divided by previous-week AUM, and returns as weekly profits relative to lagged AUM. Risk is proxied by return volatility (SD over the past 52 weeks). The sample spans 2001 July 27–2021 December. We retain economies with at least 5 years (260 weeks) of data for both sustainable and traditional investments in either equity or bond markets. The final sample includes 94 economies spanning both advanced and emerging markets across multiple continents (Table S5), accounting for 97% of world gross domestic product (GDP).

#### Climate policy

Data on carbon pricing policies (ETS and carbon taxes) are from the World Bank, including implementation dates, emissions coverage, and carbon prices. We manually collect announcement dates from official documents and news, enabling analysis of forward-looking investment responses. We consider both announcements and implementations, including policies under formal consideration or legislated adoption. By end-2021, 49 economies in our sample announced climate policies and 41 implemented them—20 economies adopted both ETS and carbon taxes, four adopted only carbon taxes, and 25 only ETS. Most policies are national; only a few (e.g. United States, Japan, and Mexico) are subnational (Table S5).

#### Climate mitigation measures

We capture policy effectiveness using three indicators: (i) carbon emissions measured by percentage change in emissions per capita between post- and prepolicy periods, normalized by prepolicy levels (World Bank WDI); (ii) renewable energy gauged by change in renewable generation normalized by prepolicy levels (42); and (iii) green innovation captured by change in green patents normalized by prepolicy levels (43).

### Methods

#### Baseline model

To identify the effect of climate policy on sustainable investment, we employ a triple-DiD design. A standard DiD compares policy-adopting and nonadopting countries before and after policy changes but may confound concurrent macroeconomic shifts. Our approach additionally contrasts sustainable and traditional investments within each country. If climate policy operates through environmental alignment, sustainable investments should increase more than traditional ones; if through general financial improvements, both should respond similarly. This within-country comparison absorbs time-varying country shocks and isolates the policy's differential effect. We estimate the following baseline model:


(1) $$Y_{a,s,i,t}=\beta \times \mathrm{SR}I_{a,s,i}\times CP_{i,t}+f_{a,s,i}+f_{a,t}+f_{i,t}+\varepsilon_{a,s,i,t},$$

where *a*, *s*, *i*, and *t*, respectively, indicate asset class (equity or bond), sustainability indicator (sustainable or traditional investments), economy, and time. The dependent variable $Y_{a,s,i,t}$ could be investment, return and risk in asset class *a* for investment type *s* in economy *i* at period *t*. The sustainability indicator, $\mathrm{SR}I_{a,s,i}$, equals 1 for sustainable investments and 0 for traditional investments in asset class *a* in economy *i*. The climate policy dummy, $CP_{i,t}$, equals 1 after the announcement of the first ETS or carbon tax, and 0 before the policy announcement and for economies that have no official plan to implement any climate policies. The asset-sustainability-economy fixed effects $f_{a,s,i}$ absorb the idiosyncratic characteristics of equity and bond markets for sustainable and traditional investments across economies, such as the cultural preference for sustainable capital, the relative dominance of green and regular products in equity and bond markets, which could potentially affect the growth, allocation, and performance of different types of investment worldwide. The asset-time fixed effects $f_{a,t}$ take care of time-varying characteristics in different asset classes such as equity and bond market returns and their associated risks. The economy-time fixed effects $f_{i,t}$ capture the time-varying country characteristics that may affect the investment decisions and performance of both sustainable and traditional investments, such as economic growth prospects, macroprudential policies, and regulations. Finally, $\varepsilon_{a,s,i,t}$ is the error term. To check the robustness of our estimation, we also employ different sets of fixed effects, alternative model specifications, and estimation techniques, and control for additional variables.

Note that the difference between sustainable and traditional investments, which should be captured by the coefficient of $\mathrm{SR}I_{a,s,i}$, is absorbed by asset-sustainability-economy fixed effects $f_{a,s,i}$. The direct impacts of climate policy on both types of investment, which should be captured by the coefficient of $CP_{i,t}$, are absorbed by economy-time fixed effects $f_{i,t}$. For example, if a climate policy improves general market sentiment or expected returns across the board, that common component is fully captured by the economy-time fixed effects.

After accounting for economy-time fixed effects, what remains in the estimated coefficient on climate policy is only the differential effect—the component that uniquely influences sustainable investments relative to traditional ones. This effect is captured by our key parameter of interest *β*, the coefficient of the interaction between sustainable investments and climate policy ($\mathrm{SR}I_{a,s,i}\times CP_{i,t}$). If climate policy increases sustainable investments, measured by the weekly capital flows to sustainable funds normalized by the asset under management of these sustainable funds, *β* should be positive and statistically significant.

#### Announcement and implementation effects

We identify the signaling effect of policy announcements and the amplification effect associated with policy enforcement by expanding Eq. 1 to include the interaction between $\mathrm{SR}I_{a,s,i}$ and ${\mathrm{CP}}_{i,t}^{\mathrm{Imp}}$, a dummy that equals 1 after the implementation of climate policy:


(2) $$\begin{array}{rl} Y_{a,s,i,t}\;= & \beta^{\mathrm{Ann}}\times \mathrm{SR}I_{a,s,i}\times CP_{i,t}+\gamma^{\mathrm{Imp}}\times \mathrm{SR}I_{a,s,i}\times {\mathrm{CP}}_{i,t}^{\mathrm{Imp}}+f_{a,s,i}\\ & +f_{a,t}+f_{i,t}+\varepsilon_{a,s,i,t} \end{array}.$$

The coefficient $\beta^{\mathrm{Ann}}$ on the first interaction term ($\mathrm{SR}I_{a,s,i}\times CP_{i,t}$) captures the signaling effect—the response of sustainable investment to climate-policy announcements prior to implementation. A positive and statistically significant $\beta^{\mathrm{Ann}}$ indicates that climate-policy announcements attract sustainable investment even before policies take effect. The coefficient $\gamma^{\mathrm{Imp}}$ of the second interaction term ($\mathrm{SR}I_{a,s,i}\times {\mathrm{CP}}_{i,t}^{\mathrm{Imp}}$) captures the additional response of sustainable investments to the implementation of climate policy on top of its announcement. If the implementation of climate policy amplifies its effect on sustainable investment, then $\gamma^{\mathrm{Imp}}$ should be positive and statistically significant.

#### Transmission channels

To test whether climate policy attracts sustainable investment through demonstrated state capacity, we expand Eq. 1 to include a triple interaction term:


(3) $$\begin{array}{rl} Y_{a,s,i,t}\;= & \beta \mathrm{SR}I_{a,s,i}\times CP_{i,t}+\rho^{\mathrm{Channel}}\mathrm{SR}I_{a,s,i}\times CP_{i,t}\times D_{i,t}^{\mathrm{Channel}}\\ & +f_{a,s,i}+f_{a,t}+f_{i,t}+\varepsilon_{a,s,i,t} \end{array}.$$

Here, $D_{i,t}^{\mathrm{Channel}}$ is a dummy that equals 1 for economies whose climate-policy-related achievements—such as reductions in carbon emissions, expansion of renewable-energy generation, and growth in green innovation—are above the sample median among countries that have formally adopted a climate policy. If more effective climate policies in mitigating and adapting to climate change attract greater sustainable investment, the coefficient on the triple interaction term, *ρ*, should be positive and statistically significant.

To test the alternative financial channel—that climate policy attracts sustainable investment by improving financial risk-return conditions—we redefine $D_{i,t}^{\mathrm{Channel}}$ as a dummy variable equal to 1 for economies where climate-policy-related reductions in sustainable investment risk or improvements in sustainable investment returns are above the sample median among countries that have formally adopted a climate policy. If climate policy fosters sustainable investment because it improves financial risk-return trade-offs, its impact should be stronger in economies with higher financial returns or lower financial risks for sustainable investment after climate policy. In this case, the coefficient on the triple interaction term, *ρ*, should be positive and statistically significant.

## Supplementary Material

pgag315_Supplementary_Data

## Data Availability

All data used in this study are publicly available with the exception of the EPFR data. The international investment flow data are obtained from Emerging Portfolio Fund Research (EPFR; https://isimarkets.com/epfr/). These data are proprietary and, under the terms of our license, cannot be publicly redistributed. Researchers interested in obtaining access to the EPFR data may contact EPFR/ISI Markets through its official data-access request page (https://isimarkets.com/epfr_demo). To support transparency and replication, a perturbed EPFR panel is provided that preserves the original structure and broad statistical properties but may not necessarily reproduce the reported results. The replication package is deposited at https://github.com/Adwenzh/PNAS_ClimateCapital_code.
