# Supplementary material for: Governing global capital: How state capacity and climate policy credibility attract sustainable investment
Source: PNAS Nexus. 2026 Oct 9;5(11):pgag315. doi: 10.1093/pnasnexus/pgag315 (PMC13729122; doi:10.1093/pnasnexus/pgag315)
Supplement: Supplementary file 1 [file pgag315_supplementary_data.pdf]

PNAS nexus

Supplementary Information for

Governing Global Capital: How State Capacity and Climate Policy Credibility Attract Sustainable Investment

Huanhuan Zheng

LI Building 5535

Department of Public and International Affairs

City University of Hong Kong

Email: H.Zheng@cityu.edu.hk

This PDF file includes:

Figure S1
Tables S1 to S5

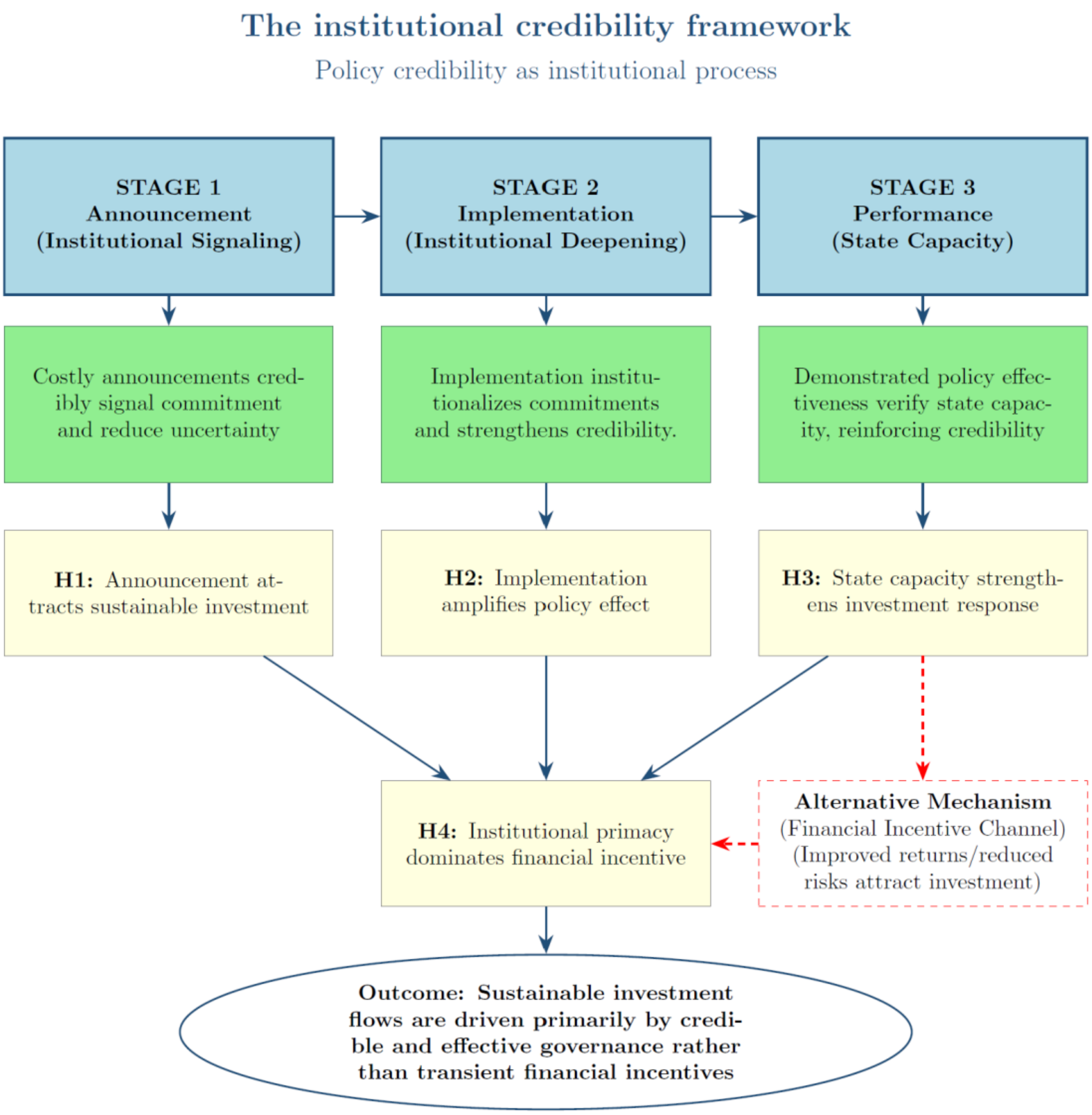

Fig. S1. Theoretical framework diagram.

Table S1. Summary statistics.

This table summarizes the mean and standard deviation (SD) of sustainable (treatment group) and traditional (control group) investments and their difference. *Investment* is the dollar amount of weekly capital flow to each economy as a ratio of its total asset under management. *Return* is the investment return, and *Risk* is the investment risk measured the standard deviation of the weekly investment returns in the past 52 weeks. *Before* and *After* refer to the periods before and after the announcement of the climate policy, respectively. *DID* is the difference between sustainable and traditional investments after the climate policy, relative to that before the climate policy. All statistics are in percentage points.

	(1)	(2)	(3)	(4)	(5)	(6)
	Sustainable		Traditional		Sustainable – Traditional	
	Mean	SD	Mean	SD	Difference	p-value
Investment						
Before	0.267	0.005	0.082	0.005	0.186	0.000
After	0.248	0.006	0.024	0.006	0.224	0.000
DID					0.038	0.000
Return						
Before	0.058	0.008	0.119	0.007	-0.061	0.000
After	0.076	0.009	0.092	0.009	-0.016	0.179
DID					0.045	0.005
Risk						
Before	1.988	0.004	1.739	0.004	0.249	0.000
After	1.792	0.005	1.756	0.004	0.035	0.000
DID					-0.214	0.000

Table S2. Robustness checks with alternative specifications.

This table reports the impact of climate policy on sustainable investments using alternative specifications of fixed effects (FE) in columns 1-6, alternative clustering at the economy-year level in column 7, Driscoll-Kraay standard errors in column 8, and Tobit estimation in column 9. The dependent variable is *Investment*, the weekly capital flows to either sustainable or traditional funds aggregated respectively over an economy normalized by the corresponding asset under management. The sustainable indicator *SRI* equals 1 for sustainable investments (treatment group), and 0 for traditional investments (control group). The post-treatment dummy *CP* equals 1 after the announcement of the climate policy, and 0 otherwise. Heterogeneity robust standard error clustered by economy is reported in the parenthesis except for columns 7 and 8. ***, **, and * denote the significance level at 1%, 5%, and 10%, respectively.

	(1)	(2)	(3)	(4)	(5)	(6)	(7)	(8)	(9)
$SRI \times CP$	0.398***	0.388***	0.389***	0.266***	0.028***	0.039***	0.388***	0.388***	0.122***
	(0.040)	(0.041)	(0.041)	(0.027)	(0.010)	(0.010)	(0.033)	(0.057)	(0.007)
SRI					0.197***	0.185***			
					(0.006)	(0.006)			
CP						-0.008			
						(0.011)			
Observations	259,524	286,533	276,995	286,595	276,961	286,561	276,961	276,961	286,595
R-squared	0.518	0.160	0.338	0.143	0.409	0.223	0.412	0.412	0.412
SRI×class×economy FE	Y	Y	Y	Y	N	N	Y	Y	N
Class×Economy×week FE	Y	N	N	N	N	N	N	N	N
Class×week FE	N	N	N	N	Y	Y	Y	Y	N
Economy×week FE	N	N	Y	N	Y	N	Y	Y	N
Class×month FE	N	Y	N	N	N	N	N	N	N
Economy×month FE	N	Y	N	N	N	N	N	N	N
Week FE	Y	N	Y	Y	Y	Y	N	N	N

Table S3. Robustness checks across asset classes.

This table reports the additional checks on the impact of climate policy on sustainable investments, differentiating equity and bond funds and controlling for additional variables. The dependent variable is *Investment*, the weekly capital flows to either sustainable or traditional funds aggregated respectively over an economy normalized by the corresponding asset under management. The sustainable indicator *SRI* equals 1 for sustainable investments (treatment group), and 0 for traditional investments (control group). The post-treatment dummy *CP* equals 1 after the announcement of the climate policy, and 0 otherwise. All regressions control for economy-time fixed effects, while column 3 control additionally for sustainability-asset-economy and asset-time fixed effects. Heterogeneity robust standard error clustered by economy is reported in the parenthesis. ***, **, and * denote the significance level at 1%, 5%, and 10%, respectively.

	(1)	(2)	(3)
Sample	*Equity*	*Bond*	*Full*
$SRI \times CP$	0.331***	0.527***	0.311***
	(0.027)	(0.079)	(0.038)
$Flow_{a,s,i,t-1}$			0.083***
			(0.009)
$Return_{a,s,i,t-1}$			0.052***
			(0.007)
$Flow\ volatility_{a,s,i,t-1}$			-0.028**
			(0.012)
$Return\ volatility_{a,s,i,t-1}$			-0.142***
			(0.020)
Observations	136,706	122,818	258,532
R-squared	0.517	0.517	0.523

Table **S4**. Stringency of climate policy.

This table summarizes how sustainable investments respond to different types of climate policy representing various levels of carbon regulation stringency. The dependent variable is *Investment*, the weekly capital flows to either sustainable or traditional funds aggregated respectively over an economy normalized by the corresponding asset under management. The sustainable indicator *SRI* equals 1 for sustainable investments (treatment group), and 0 for traditional investments (control group). The post-treatment dummy *CP* equals 1 after the announcement of the climate policy, and 0 otherwise. The dummy variables $D^{HighCarbonPrice}$ and $D^{HighCarbonTax}$ equal 1 respectively if the carbon price and tax are above the sample median. The dummy variable $D_{i,t}^{Subnational}$ ($D_{i,t}^{Both}$) equals 1 if the economy rolls out climate policy at only subnational (both subnational and national) level. All regressions control for sustainability-asset-economy, asset-time, and economy-time fixed effects. Heterogeneity robust standard error clustered by economy is reported in the parenthesis. ***, **, and * denote the significance level at 1%, 5%, and 10%, respectively.

	(1)	(2)	(3)
$SRI \times CP$	0.228***	0.456***	0.399***
	(0.047)	(0.052)	(0.046)
$SRI \times CP \times D^{HighCarbonPrice}$	0.202***		
	(0.033)		
$SRI \times CP \times D^{HighCarbonTax}$		-0.087	
		(0.059)	
$SRI \times CP \times D^{Subnational}$			-0.313***
			(0.058)
$SRI \times CP \times D^{Both}$			0.221*
			(0.113)

Table S5. List of economies. Panel A lists the economies that had announced to implement either emission trading system (ETS) or carbon tax to regulate carbon emissions by the end of 2021. The economies that have announced but not yet implemented either ETS or carbon taxes is marked in *Italic* and red. The economies that have only implemented the climate policy at subnational level is highlighted in **bold**. Panel B lists the economies that had not yet adopted climate policies

**A. Countries with climate policy**				
**ETS only**		**Both ETS & carbon tax**		**Carbon tax only**
Australia	Kazakhstan	Canada	Netherlands	Argentina
Austria	Lithuania	*Chile*	Poland	Norway
Belgium	New Zealand	*Colombia*	Portugal	Singapore
Bulgaria	*Pakistan*	Denmark	Slovenia	South Africa
China	Romania	Estonia	Spain	
Croatia	*Russia*	Finland	Sweden	
Cyprus	*Serbia*	France	Switzerland	
Czech Republic	South Korea	Ireland	**United States**	
Germany	*Taiwan*	**Japan**	Ukraine	
Greece	*Thailand*	**Mexico**	United Kingdom	
Hungary	*Turkey*			
Indonesia	*Vietnam*			
Italy				

**B. Countries without climate policy**				
Azerbaijan	Egypt	Israel	Mauritius	Saudi Arabia
Bahrain	Ethiopia	Jamaica	Morocco	Sri Lanka
Bangladesh	Gabon	Jordan	Myanmar (Burma)	Tanzania
Bosnia Herzegovina	Georgia	Kenya	Nigeria	Trinidad and Tobago
Brazil	Ghana	Kuwait	Oman	Tunisia
Congo	Honduras	Lebanon	Panama	United Arab Emirates
Cuba	Hong Kong SAR	Macedonia	Peru	Venezuela
Dominican Republic	India	Malawi	Philippines	Zambia
Ecuador	Iraq	Malaysia	Qatar	Zimbabwe
